# 6-year follow-up on migration outcomes: a randomised clinical trial of cemented vitamin E-stabilised highly crosslinked versus standard polyethylene cup in total hip arthroplasty

**DOI:** 10.1177/11207000241267971

**Published:** 2024-09-18

**Authors:** Olof Sköldenberg, Sebastian Mukka, Martin Magneli

**Affiliations:** 1 Department of Clinical Sciences at Danderyd Hospital, Unit of Orthopaedics, Karolinska Institute, Stockholm, Sweden; 2 Department of Surgical and Perioperative Sciences (Orthopaedics), Umeå University, Sweden

**Keywords:** Total hip replacement, Radiostereometric analysis, RSA, Randomized clinical trial, Vitamin E Highly Crosslinked Polyethylene, Highly Crosslinked Polyethylene

## Abstract

**Background:**

In a previous study we have shown that a cemented vitamin E-doped highly cross-linked polyethylene (VEPE) compared to a conventional polyethylene cup in total hip arthroplasty (THA) has a slightly higher proximal migration but significantly lower wear rates up to 2 years after surgery. In this follow-up study we investigated the same cohort at 6 years.

**Methods:**

This was a double-blinded, non-inferiority, randomised controlled trial on patients with osteoarthritis, with a mean age of 66 years. Patients were randomly assigned to receive either the conventional polyethylene cup or the VEPE cup in a 1:1 ratio. The primary endpoint was proximal implant migration of the cup measured with radiostereometric analysis (RSA). Secondary endpoints included wear rate of the cup and patient-reported outcome measurements (PROM).

**Results:**

At the 6-year follow-up, 25 patients (11 controls, 14 VEPE) were available for RSA measurements, and we found no statistically significant difference in proximal migration between the VEPE and control groups. The wear rate was significantly lower in the VEPE group compared to controls, 0.03 mm/year and 0.07 mm/year, respectively with a mean difference 0.04 mm, (95% CI, 0.02–0.06 mm). There were no cup revisions and no difference in PROM between the groups.

**Conclusions:**

Based on our 6-year results, the VEPE group exhibited no statistical or clinically relevant difference compared to the control group, and the wear rate was significantly lower in the VEPE group. The use of a cemented vitamin E-doped highly cross-linked cup is a good option in total hip arthroplasty.

## Introduction

Vitamin E-doped, hig2hly-crosslinked polyethylene (VEPE) has emerged as a promising alternative to conventional ultra-high molecular weight polyethylene (UHMWPE) in total hip arthroplasty (THA) due to its superior wear characteristics and resistance to oxidative stress.^1^ Our research group previously conducted the first randomised radiostereometric (RSA) clinical trial on VEPE in cemented cups used in THA,^2–4^ which demonstrated low head penetration rates up to 2 years postoperatively but a higher migration rate of the acetabular component when compared to controls.^5^

While uncemented THA has shown promise with the use of VEPE, bone cement fixation remains the most prevalent method for securing the acetabular component in many countries, including Sweden.^6^

Consequently, the current study is a mid-term follow-up study which aims to evaluate the performance of a VEPE implant in comparison to a well-documented, geometrically identical cemented cup of conventional PE at 6 years.

## Patients and methods

### Setting, design and participants

This study is a 6-year follow-up of a previously published single-centre, randomised, double-blind, non-inferiority trial with inclusion period 2013-2015 at the Orthopaedic Department of Danderyd Hospital, Stockholm.^5,7^ The original study included patients between the ages of 40 and 75 years with hip osteoarthritis, and this follow-up examines those who had participated in the 2-year follow-up. Study data were managed using REDCap electronic data capture tools.^8^

### Sample size

The original study aimed to analyse 42 participants with complete radiostereometric analysis (RSA) data at the 2-year follow-up. The study was designed to show that the VEPE group, compared with the control group, had neither lower nor higher proximal migration (y-translation) than the clinically relevant migration threshold of 0.2 mm,^2^ in effect demonstrating that the VEPE was not inferior to the standard PE. The sample for the current study was drawn from the original study cohort using a convenience sampling method, comprising all available patients for whom 6 years had elapsed since surgery.

### Randomisation, blinding, and intervention

We used concealed envelopes to randomly assign patients in a 1:1 ratio to either the control or VEPE group, with sex and age (<65 and ⩾65 years) as stratification factors. Treatment was blinded to all parties except the surgeons, as the vitamin-E implants were slightly yellow. RSA analysis and radiographic evaluation were performed blinded to allocation. A posterolateral approach was used and the cemented acetabular component used was the Exceed ABT Cemented Cup (Zimmer-Biomet), with identical geometry for both groups, consisting of either VEPE or an argon-gas gamma-sterilised PE (control group, ArCom). A third-generation cementation technique with 6–8 anchoring holes and cement compression (Refobacin, Zimmer-Biomet) was used. The femoral component was a Bi-Metric HA titanium alloy stem with a 32-mm chromium-cobalt head. All surgeons had long experience with the implants and a standard fast-track protocol was used for rehabilitation.

### Outcome measurements

The primary endpoint was proximal migration (y-translation) of the cup, measured with RSA. This endpoint was chosen since every mm increase in 2-year proximal migration has been verified to increase the risk of revision of an acetabular implant by 10% at 10 years.^2^ Even a small mean translation of over 0.2 mm at 2 years suggests a 5% increased risk of revision due to aseptic loosening. This predictive ability of early micromotion is currently widely accepted in RSA studies and set as gold standard in evaluating acetabular implants in hip arthroplasty.^2–4,9^

The secondary endpoints included total migration of the cup and head penetration of the femoral head into the cup. Patient-reported outcome measurements (PROMS) and adverse events were recorded preoperatively and at follow-ups. RSA were done according to published guidelines, with tantalum-markers placed during surgery and RSA measurements taken at various intervals. The precision of our RSA setup was comparable to previous reports.

### Statistical analyses

All patients allocated to either group were included in the analyses, based on the intention-to-treat principle. Data were tested for normality using the Shapiro-Wilk test and they were not normally distributed, therefore the The Mann-Whitney U-test was used to detect statistically significant difference between the groups. Fisher’s Exact test was used to evaluate the proportion of components with a proximal migration of over 0.2 and 1.0 mm in relation to the study group. The analyses were done with SPSS 23.0 (SPSS, Chicago, IL, USA).

## Results

**Figure 1.**
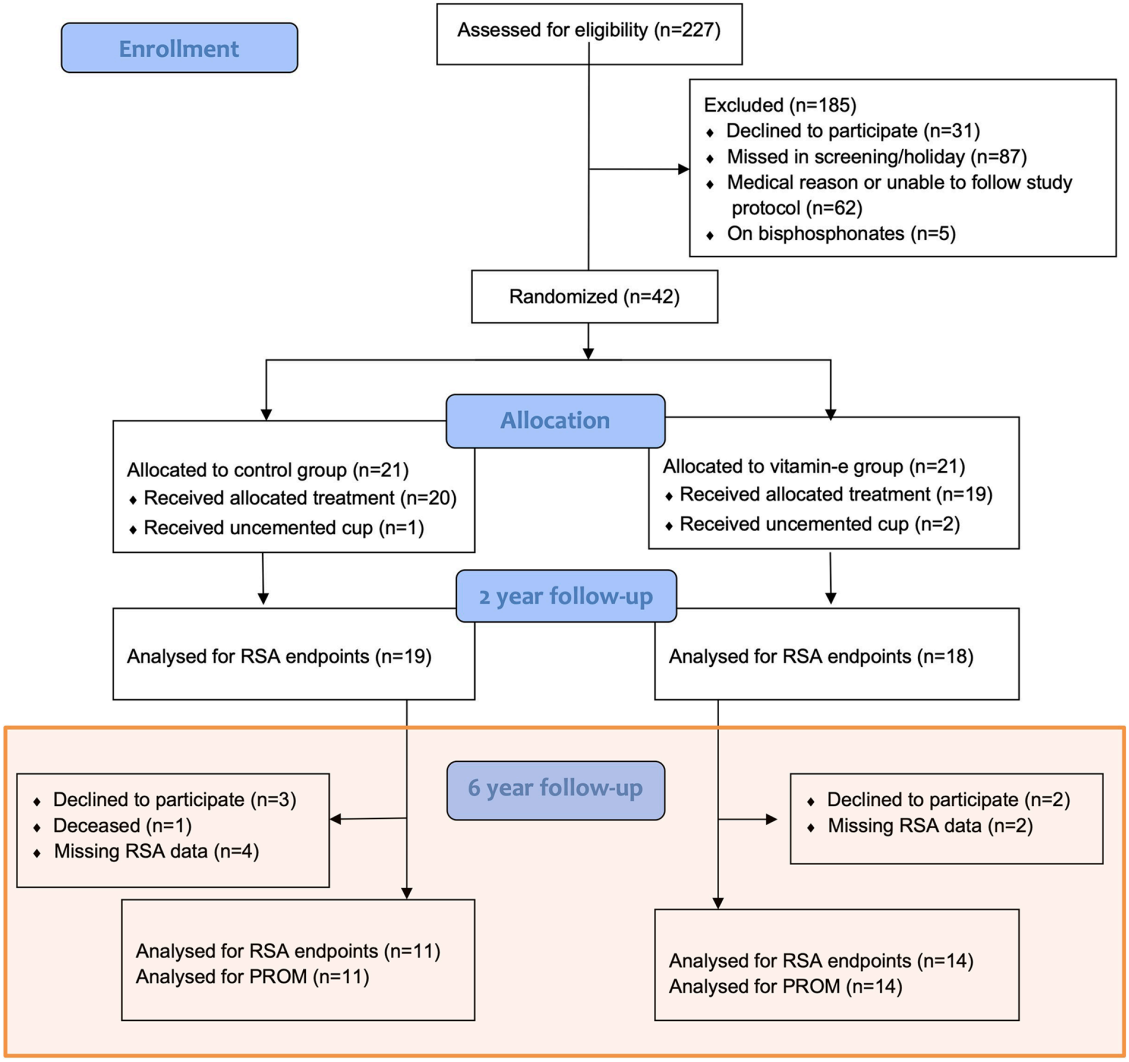
Flow of patients in accordance with CONSORT (Consolidated Standards of Reporting Trials).

Out of 42 eligible study participants, 25 patients (mean age 66 years, male: females, 14:11) were analysed for the primary endpoint at 6 years (11 control group and 14 VEPE group) (Table 1) (Figure 1).

**Table 1.** Baseline characteristics of the 25 patients in the 6-year follow-up study.

	Control group (*n* = 11)	VEPE group (*n* = 14)
Age at primary surgery, yrs^a^	66 ± 5	66 ± 5
Sex^b^		
Female	6 (55)	8 (57)
Male	5 (45)	6 (43)
Weight, kg^a^	80 ± 12	81 ± 16
Height, cm^a^	172 ± 8	175 ± 10
BMI, kg/m^2a^	28 ± 4	27 ± 4
ASA classification^b^		
1–2	10 (91)	11 (79)
3–4	1 (9)	3 (10)
Charnley class^b^		
A	6 (55)	10 (71)
B	4 (36)	3 (21)
C	1 (9)	1 (7)
Operated side^b^		
Right	7 (64)	7 (50)
Left	4 (36)	7 (50)
Preop Harris hip score^a^	45 ± 16	48 ± 16
Preop EQ-5D index^a^	0.4 ± 0.3	0.5 ± 0.3

BMI, body mass index; ASA, American Society of Anesthesiologists.

a mean ± standard deviation (SD).

b *n* (%).

### Primary endpoint

**Figure 2.**
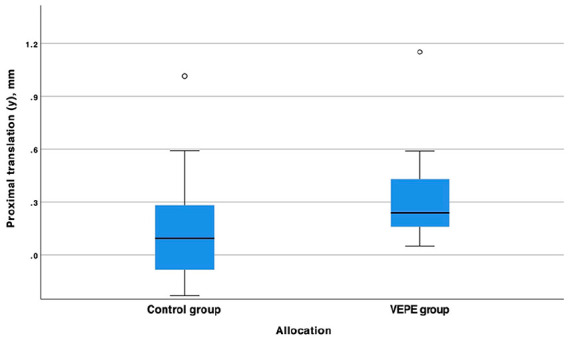
Box plot of the proximal translation (y-axes) of the cemented cup in relation to the acetabulum in the control and VEPE group. We found no statistically significant difference between the groups.

At the 6-year follow-up, the VEPE group exhibited a proximal median (range) migration (y-translation) of 0.24 mm (0.05–1.15 mm) compared to the control group, which had a median migration of 0.09 mm (−0.23–1.02 mm). This difference was not statistically significant, or clinically relevant (Table 2) (Figure 2). In addition, a total of 9 cups in the VEPE group exceeded the proposed safe threshold of 0.2 mm for proximal migration compared to the control group (7 cups), this difference was not statistically significant. 1 cup in each group had a migration >1.0 mm at 6 years.

**Table 2.** Primary and secondary endpoints at the 6-year follow-up. Migration data of components measured with RSA at 6 years.

	Control group				VEPE group		
	Median	Min	Max	Median	Min	Max	*p*-Value
**Primary endpoint**							
Proximal translation (y), mm	0.09	−0.23	1.02	0.24	0.05	1.15	
**Secondary endpoints**							
Total translation (3D), mm	0.31	0.12	1.25	0.42	0.13	1.37	
Transverse translation (x), mm	0.12	−0.85	0.26	0.15	−0.32	0.61	
Anteroposterior translation (z), mm	−0.03	−0.69	0.16	−0.11	−0.64	0.40	
Flexion/extension rotation (x), °	0.05	−0.54	1.47	−0.07	−1.01	1.19	
Ante-, retroversion (y), °	0.11	−1.52	0.88	−0.22	−0.78	1.20	
Varus/valgus rotation (z), °	0.01	−2.68	1.84	−0.06	−1.61	2.80	
***Wear data***							
Medial femoral head penetration (x), mm	−0.06	−0.19	0.19	0.02	−0.14	0.19	
Proximal femoral head penetration (y), mm	0.36	0.00	0.75	0.03	−0.03	0.16	<0.001
Anterioposterior femoral head penetration (z), mm	−0.04	−0.18	0.54	0.03	−0.61	0.11	
Total femoral head penetration (3D), mm	0.39	0.14	0.92	0.15	0.04	0.62	<0.001
***PROM data***							
Harris Hip Score	94	63	100	95	60	100	
EQ-5D index	0.9	0.5	1.0	1.0	0.7	1.0	

All migration values are compared to the postoperative baseline RSA measurements. *P*-values are derived from the Mann-Whitney U-test and presented if <0.05.

### Secondary endpoints

**Figure 3.**
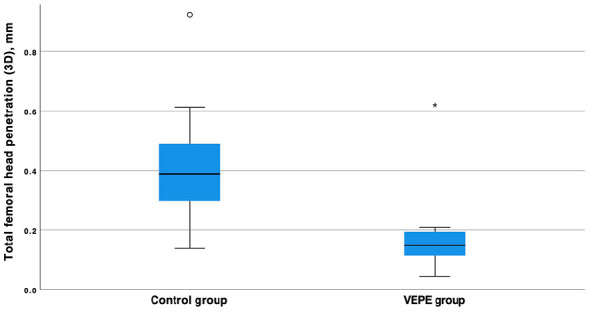
Box plot of femoral head penetration of the femoral head in relation to the cup in the control group and VEPE group. The difference between the groups is statistically significant with a *p*-value of <0.001 (Mann-Whitney U-test).

In the secondary migration endpoints, the femoral head penetration was statistically significantly lower in the VEPE group with a median (range) femoral head penetration of 0.15 mm (0.04–0.62) compared to the control group, which had a median femoral head penetration of 0.39 mm (0.14–0.92, *p*-value <0.001 Mann-Whitney U-test) (Table 2) (Figure 3). The annual wear rate was significantly lower in the VEPE group compared to controls, 0.03 mm/year and 0.07 mm/year, respectively with a mean difference 0.04 mm, (95% CI, 0.02–0.06 mm).

The radiolucent zones in DeLee Charnley zones remained unchanged in comparison to the postoperative radiographs in 5 out of 11 controls and in 6 out of 14 VEPE implants respectively and remained similar between the groups. No additional zones developed between the 2-year and 6-year follow-up in either group. We found no correlation between development of radiolucency in Charnley DeLee zones and overall migration of the cup or femoral head penetration. Prior to the 2-year follow-up, each group had 1 instance of component revision due to recurrent dislocations. Subsequently, at the 6-year follow-up, neither group experienced any revisions of the acetabular component, and no cups were deemed clinically unstable.

The PROM outcome including Harris Hip Score (HHS) and EQ-5D index did not differ between the groups with no statistically or clinically different between the groups (Table 2).

### Patients with missing RSA data

At the 6-year follow-up, RSA data were available for 25 out of the initial 42 patients. The reasons for missing data included patient refusal to participate, 1 deceased patient and technical issues during RSA measurements (Figure 1). There were no statistically significant differences in PROM data between patients with and without RSA data. None of the 17 patients with missing RSA data required revisions or other reoperations.

## Discussion

The presented mid-term results still show no statistically or clinically relevant difference in proximal cup migration between the 2 groups. Furthermore, we observed, as expected with VEPE, a significantly lower femoral head penetration and annual wear rate compared to controls.

### Strengths and limitations

This study was a prospective, double-blinded, randomised controlled trial that adhered to a strict study protocol,^7^ which lends validity to our findings. Our study was conducted under regular clinical conditions in a standard cohort of osteoarthritis patients, and the surgeries were performed by experienced hip arthroplasty surgeons who routinely use cemented acetabular components.

A major limitation of our study is the small sample size available at 6 years, which limits our ability to detect small differences in migration rates between the two groups. Furthermore comparisons from RSA data of the original 2- year follow-up are difficult as the study now only evaluated 25 of the original 42 patients included. An analysis of the clinical outcome PROM data showed no significant differences between patients with available RSA data and those without. This suggests that the missing data did not introduce any systematic bias into the study’s outcomes. Consequently, we consider the results to be representative of the initial study cohort. Finally, as our study was conducted in a single centre, our findings may not be generalisable to other settings or populations.

## Generalisability

Our findings at 2 years follow-up revealed a worrisome trend of small, yet continuous proximal migration combined with increasing abduction angle in the VEPE group,^5^ which has previously been a harbinger of aseptic loosening in other cemented implants. Furthermore, we observed radiolucent zones in both implant groups, with their distributions over size, location and progression rate not being statistically different. However, at this 6-year follow-up, we did not detect any significant difference in proximal migration between the 2 groups, with the clinical results being identical and no cup revisions in either group. Moreover, the wear rate between the groups significantly favoured the VEPE group, with a lower femoral head penetration rate compared to the control group as has been shown in several other studies on this topic.^10–13^ Our RSA data were similar to a newly published study by Bergvinsson et al.^14^ In randomised clinical trial on identical acetabular components on 48 patients, they found low wear rates for the VEPE group, had a tendency towards higher migration of the VEPE group at 3 months and 1 year but at 5 years had identical migration patterns in both groups.

Our study underscores the importance of long-term studies for evaluating new implant technologies’ safety and efficacy, highlighting radiostereometric analysis (RSA) as a critical method for tracking implant migration.^2^ CT-RSA, an advancement over traditional RSA that uses computed tomography (CT) scanners, simplifies the process without requiring implant modifications or metal markers. While CT-RSA enhances accessibility for researchers, clinical follow-up and arthroplasty registry monitoring remain indispensable for comprehensive assessment.

The development of new implant technology should be introduced in a step-wise fashion, and clinical implementation should be preceded by trials comparing them to well-functioning and well-documented implants^15^ Modifications of implant designs, materials or production process can have substantial adverse effects on implant survival, and such modifications are continuously being made today, as in the case of vitamin-E doped highly crosslinked polyethylene with the scientific hypothesis primarily to reduce wear and embrittlement by improved oxidative stability.^1,16^ With the large number of recent published clinical trials and cohort studies on the positive (i.e. low) wear characteristics of VEPE-implants, they now have the potential to further prevent osteolysis and implant loosening even in the long-term.^14,17–19^ The VEPE implant in the present study exhibit similar proximal femoral head penetration as a widely used highly cross-linked all-polyethylene cup.^19^

Our study provides important insights into the generalisability and safety of new implant technology, specifically vitamin-E doped highly cross-linked polyethylene cups. Although our initial RSA results were concerning, we did not detect any significant difference in proximal migration between the VEPE and control groups at 6 years.

## Conclusion

At 6-year follow-up the VEPE group exhibited no significant difference in proximal migration compared to the control group, and the wear rate was significantly lower in the VEPE group. The presented RSA data support the use of a vitamin E-doped highly cross-linked cup as a good option in cemented total hip arthroplasty.
